# Obituary

**Published:** 1881-07

**Authors:** 


### OBITUARY.

Died in this city, of heart disease, on Sabbath, June 12th, 1881, at 10:30 o'clock, p. m., Dr. James Taylor, M. D., D. D. S., in the 72d year of his age. Some biographical mention of the Doctor will be made in the next number of the Register.

